# Antiepileptic treatment may determine the outcome of *FARS2* mutation carriers

**DOI:** 10.1016/j.ymgmr.2018.09.004

**Published:** 2018-10-01

**Authors:** Josef Finsterer, Fulvio A Scorza, Carla A Scorza

**Affiliations:** a Krankenanstalt Rudolfstiftung, Messerli Institute, Veterinary University of Vienna, Vienna, Austria; b Disciplina de Neurociência. Escola Paulista de Medicina/Universidade Federal de São Paulo/. (EPM/UNIFESP). São Paulo, Brasil

**Keywords:** Mitochondrial, Genetics, Epilepsy, Antiepileptic FARS2, Phenotype

Letter to the Editor

The article by Almannai et al. about a cohort of 15 patients with FARS2 deficiency is interesting but has a number of shortcomings.

It is reported that in 11/15 patients with epilepsy, seizures were difficult to control [1]. Most of the patients received levetirazetam, phenobarbital (PB), clobazam, or lacosamide. Some patients also received valproic acid (VPA) [1]. It is well acknowledged that seizures in mitochondrial disorders maybe enhanced by mitochondrion-toxic antiepileptic drugs (AEDs), such as VPA, PB, carbamazepine, or phenytoin [2]. For understanding the outcome and phenotype it is essential to know how many patients received mitochondrion-toxic AEDs in mono- or polytherapy. Patients under VPA or PB might profit from replacing these AEDs. Additionally, we propose to try the ketogenic diet, particularly in patients in whom seizures are not well controlled. In some cases with mitochondrial epilepsy, ketone bodies may be the only effective treatment [3].

It is reported that all patients had developed visual impairment with normal eyeground and without optic atrophy [1]. It would be worthwhile to mention how many patients had abnormal visually-evoked potentials [4] or abnormal intra-cerebral visual pathways on imaging. Was elevated cerebral lactate the culprit?

There is a discrepancy between cerebral lactate elevation on MRS and by direct measurement lactate in the CSF in patient-1 [1]. Patient-1 had no lactate-peak on MRS but elevated CSF lactate [1]. No explanations were provided for this discrepancy.

MRI studies revealed bilateral DWI hyperintensities in the tegmental tract, and the parietal, occipital, and temporal cortex [1]. We should be informed if DWI-hyperintensities were accompanied by hypointensities (zytotxic edema) or hyperintensities (vasogenic edema) on ADC. Is it conceivable that some patients had stroke-like episodes?

In summary, this study may profit from replacing mitochondrion-toxic AEDs, from application of the ketogenic diet, from recording VEPs, and from revision of the MRIs.

## Conflicts of interest

There are no conflicts of interest.

## Funding

No funding was received.

## References

[1] Almannai M., Wang J., Dai H., El-Hattab A.W., Faqeih E.A., Saleh M.A., Al Asmari A., Alwadei A.H., Aljadhai Y.I., Alhashem A., Tabarki B., Lines M.A., Grange D.K., Benini R., Alsaman A.S., Mahmoud A., Katsonis P., Lichtarge O., Wong L.C. (2018 Jul 29). FARS2 deficiency; new cases, review of clinical, biochemical, and molecular spectra, and variants interpretation based on structural, functional, and evolutionary significance. Mol. Genet. Metab..

[2] Finsterer J., Zarrouk Mahjoub S. (2012). Mitochondrial toxicity of antiepileptic drugs and their tolerability in mitochondrial disorders. Expert Opin. Drug Metab. Toxicol..

[3] Kayyali H.R., Gustafson M., Myers T., Thompson L., Williams M., Abdelmoity A. (2014). Ketogenic diet efficacy in the treatment of intractable epileptic spasms. Pediatr. Neurol..

[4] Finsterer J. (2001). Visually evoked potentials in respiratory chain disorders. Acta Neurol. Scand..

